# Tipping the Balance: *Sclerotinia sclerotiorum* Secreted Oxalic Acid Suppresses Host Defenses by Manipulating the Host Redox Environment

**DOI:** 10.1371/journal.ppat.1002107

**Published:** 2011-06-30

**Authors:** Brett Williams, Mehdi Kabbage, Hyo-Jin Kim, Robert Britt, Martin B. Dickman

**Affiliations:** Institute for Plant Genomics and Biotechnology, Department of Plant Pathology and Microbiology, Texas A&M University, College Station, Texas, United States of America; Virginia Polytechnic Institute and State University, United States of America

## Abstract

*Sclerotinia sclerotiorum* is a necrotrophic ascomycete fungus with an extremely broad host range. This pathogen produces the non-specific phytotoxin and key pathogenicity factor, oxalic acid (OA). Our recent work indicated that this fungus and more specifically OA, can induce apoptotic-like programmed cell death (PCD) in plant hosts, this induction of PCD and disease requires generation of reactive oxygen species (ROS) in the host, a process triggered by fungal secreted OA. Conversely, during the initial stages of infection, OA also dampens the plant oxidative burst, an early host response generally associated with plant defense. This scenario presents a challenge regarding the mechanistic details of OA function; as OA both suppresses and induces host ROS during the compatible interaction. In the present study we generated transgenic plants expressing a redox-regulated GFP reporter. Results show that initially, *Sclerotinia* (via OA) generates a reducing environment in host cells that suppress host defense responses including the oxidative burst and callose deposition, akin to compatible biotrophic pathogens. Once infection is established however, this necrotroph induces the generation of plant ROS leading to PCD of host tissue, the result of which is of direct benefit to the pathogen. In contrast, a non-pathogenic OA-deficient mutant failed to alter host redox status. The mutant produced hypersensitive response-like features following host inoculation, including ROS induction, callose formation, restricted growth and cell death. These results indicate active recognition of the mutant and further point to suppression of defenses by the wild type necrotrophic fungus. Chemical reduction of host cells with dithiothreitol (DTT) or potassium oxalate (KOA) restored the ability of this mutant to cause disease. Thus, *Sclerotinia* uses a novel strategy involving regulation of host redox status to establish infection. These results address a long-standing issue involving the ability of OA to both inhibit and promote ROS to achieve pathogenic success.

## Author Summary

Necrotrophic fungal pathogens need to kill plant cells to establish disease and obtain nutrition. While such pathogens are economically important, they are relatively understudied and mechanistic details important for pathogenic success are limited. *Sclerotinia sclerotiorum* is a necrotrophic ascomycete fungus that infects virtually all dicotyledonous (>400 species) plants. Our data indicate that oxalic acid production and modulation of reactive oxygen species (ROS) are key components for the successful interaction of this fungus with the host plant. Here, we use a GFP regulated reporter system to analyze the host redox status during infections with wild type and a non-pathogenic oxalic acid (OA) deficient *Sclerotinia* mutant. Additionally, we show that secreted OA enables *Sclerotinia* to hijack the host cell redox machinery, initially creating reducing conditions followed by an oxidizing environment that is necessary for pathogenesis. We also provide evidence that the OA-deficient mutants are actively recognized by the plant resulting in the elicitation of a hypersensitive-like response and resistance. Our study provides insight into how *Sclerotinia*, and quite possibly other necrotrophic pathogens, co-opt host redox and cell death pathways for successful colonization of the host.

## Introduction

*Sclerotinia sclerotiorum* is a devastating and economically important necrotrophic fungal pathogen capable of infecting more than 400 species of dicotyledonous plants worldwide [1], [2] causing annual crop losses exceeding $200 million in the United States alone [2]. Diseases caused by *S. sclerotiorum* are responsible for considerable damage, have proven difficult to control (culturally or chemically), and host genetic resistance to this fungus has been inadequate (http://www.ars.usda.gov/Research/docs.htm?docid=20320&page=1). Necrotrophic plant pathogens require dead host tissue in order to obtain nourishment. Traditionally, the resulting disease symptoms have been attributed to direct killing of host tissue via secretion of toxic metabolites by the pathogen. Recently however, emerging data from several pathosystems have suggested that necrotrophic fungi are tactically more subtle in the manner by which pathogenic success is achieved, though the mechanistic details are not known. Consistent with other necrotrophs, *S. sclerotiorum* produces a wide array of degradative lytic enzymes (e.g. endo, exo-pectinase, cellulase, hemicellulase, protease), which are believed to facilitate colonization and host cell wall degradation [3], [4]. We have been investigating the role of fungal secreted oxalic acid (OA) in pathogenicity of *S. sclerotiorum* [5]–[9]. OA (dicarboxylic acid) is remarkably multifunctional and contributes to numerous physiological processes (e.g. reduction in pH, acidity-induced activation of enzymes, elevation of Ca^2+^, guard cell regulation, vascular plugging with oxalate crystals) that augment fungal colonization of host plants (reviewed in [10]). Additionally, studies with OA-deficient mutants strongly suggest that OA is an essential pathogenicity determinant and a key factor governing the broad pathogenic success of this fungus [2], [5], [11]. We have shown that OA is a fungal elicitor that induces cell death in host plant tissue resulting in hallmark apoptotic-like features including cell shrinkage, DNA laddering, and TUNEL reactive cells in a time and dose dependent manner. Oxalic acid also aids *Sclerotinia* pathogenicity indirectly acting as a signaling molecule, via manipulation of host ROS [12].

Reactive oxygen species have long been considered detrimental to cells since they can be toxic; causing damage to proteins, lipids (membranes) and nucleic acids. Recent data however suggests a more subtle and versatile role for these small molecules. When present at low levels, ROS may actually be beneficial, serving as secondary messengers in intra and inter-cellular signaling pathways. Regulation of redox homeostasis is now an active area of research, particularly within pathogen/host interactions (e.g. hypersensitive response and the oxidative burst) and adaptation to abiotic stress (e.g. drought, salt), all of which have strong correlations with ROS signaling.

One of the earliest and most universal resistance responses mounted by plant tissues against an invading microbe is the oxidative burst, a controlled release of O_2_^−^ and H_2_O_2_ at the point of pathogen challenge. Once triggered, the oxidative burst is believed to be required for pathogen defense and is expressed in almost all plant species [13]. Additionally, the oxidative burst also occurs during compatible interactions, but the timing and magnitude differ. Previous studies have shown that the oxidative burst can be suppressed at low pH [14]. As such, the release of oxalate could enhance fungal pathogenicity by acidifying host cells and dampening the oxidative burst. In this paper, we provide real-time evidence that this potent necrotroph modulates host-PCD pathways through secretion of OA, by a mechanism independent of acidification. Surprisingly, this process is initiated by a reducing environment generated by the fungus in host cells. As a direct consequence of this redox manipulation, the fungus subverts host defense responses, inhibits the oxidative burst, and prepares the infection court for the establishment of disease. Moreover, OA^−^ (non-pathogenic) mutants are unable to suppress plant defense resulting in active recognition of the fungus by the plant that is accompanied by delimited cell death and callose formation suggestive of an HR-like response.

## Results

### Plants challenged with an OA-deficient *Sclerotinia* mutant (A2) exhibit a phenotype reminiscent of a plant hypersensitive response

**Figure 1:**
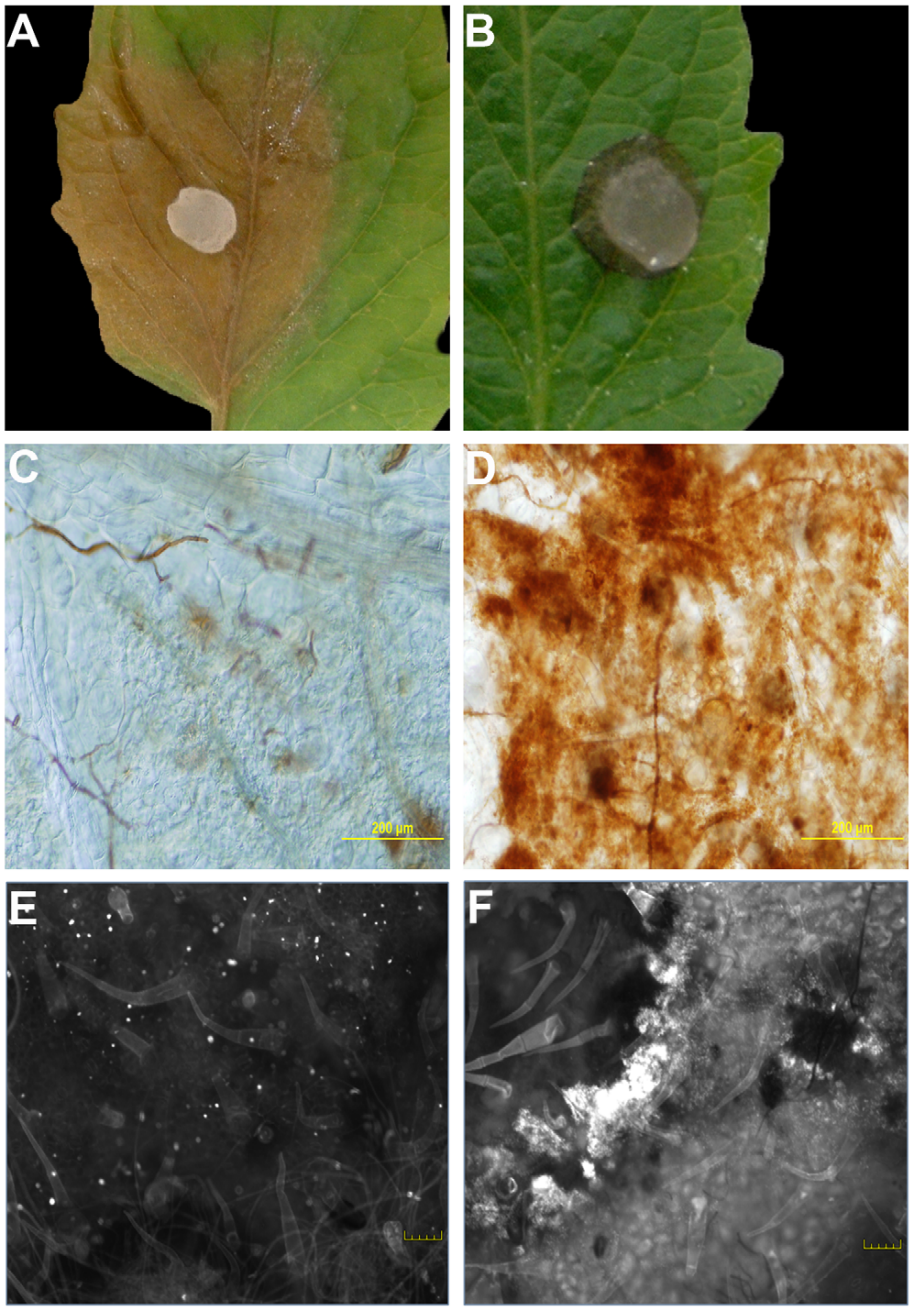
The oxalic acid (OA) deficient mutant strain (A2) induces an HR-like response. Agar plugs containing actively growing cultures of *Sclerotinia sclerotiorum* were inoculated onto tomato leaves. Wild-type (A) was pathogenic and induced runaway cell death; however, growth of the A2 strain (B) was restricted by the plant. Pictures were taken 48 hours post-inoculation. Markers associated with the HR were observed specifically following mutant, but not wild type inoculations. Using DAB staining, the oxidative burst was visible in response to the A2 strain (D), but not the wild-type (C) 8 hours post-inoculation. Unlike the wild-type (E), the A2 strain also induced callose deposition (F). Callose deposition was evaluated after 2 days. Both callose deposition and DAB staining are shown at the leading edge of the lesions.

Previously we showed that OA, secreted by *Sclerotinia*, is a pathogenicity determinant and elicitor of plant programmed cell death [12]. Consistent with these observations, we noted an intriguing difference between the disease phenotypes of wild type and OA-deficient A2 *Sclerotinia* infected plants (Figure 1). In contrast to wild type *Sclerotinia*, which causes overwhelming disease and runaway cell death. Challenge with the non-pathogenic OA-deficient A2 mutant resulted in restricted growth, reminiscent of an HR-like response (Figure 1A, 1B). To determine whether this response had features consistent with the HR, we examined several markers associated with the HR. Plant defense responses involving HR are typified by the oxidative burst, a universal and early response by the plant upon recognition of pathogens. Plants were challenged with the wild type and A2 mutant; 8 hrs post-inoculation leaves were stained with the ROS (hydrogen peroxide) indicator stain, 3,3′-diaminobenzidine (DAB). Interestingly, ROS was virtually absent in DAB stained leaf tissue challenged with wild type *Sclerotinia*, even though disease progression was observed (Figure 1C). However, leaves inoculated with the A2 mutant displayed strong DAB staining surrounding the infection point (Figure 1D).

Plants protect themselves using both physical and chemical defenses. Callose is an effective barrier induced at the site of attack during the early stages of pathogen invasion and is an established marker associated with incompatible (HR) responses. Aniline blue staining was used to reveal callose structures in leaf tissue following inoculation with wild type and the A2 mutant. Similarly to DAB, callose deposition was observed following inoculation with the A2 mutant but not wild type (Figure 1E, 1F). Thus markers associated with plant defense were observed specifically following mutant, but not wild type inoculations; including the oxidative burst and callose deposition. It is of interest to determine whether the phenotype induced in response to the A2 mutant mechanistically resembles an HR, as there are no known proteinaceous effectors or corresponding resistance (R) proteins in this system. Such results would suggest active recognition of the A2 strain by the host, followed by an effective host defense response which does not occur in response to challenge with the wild type fungus. In contrast, the A2 mutant could be just physiologically compromised in pathogenicity and thus incapable of causing disease independent of plant involvement. Furthermore, these findings also suggest that the wild type fungal strain suppresses this recognition process. Existing models describing the mechanisms of recognition and response to pathogens have been, for the most part, centered on plant interactions with biotrophs.

### *Sclerotinia* suppresses the host oxidative burst by modulation of the host redox environment

**Figure 2:**
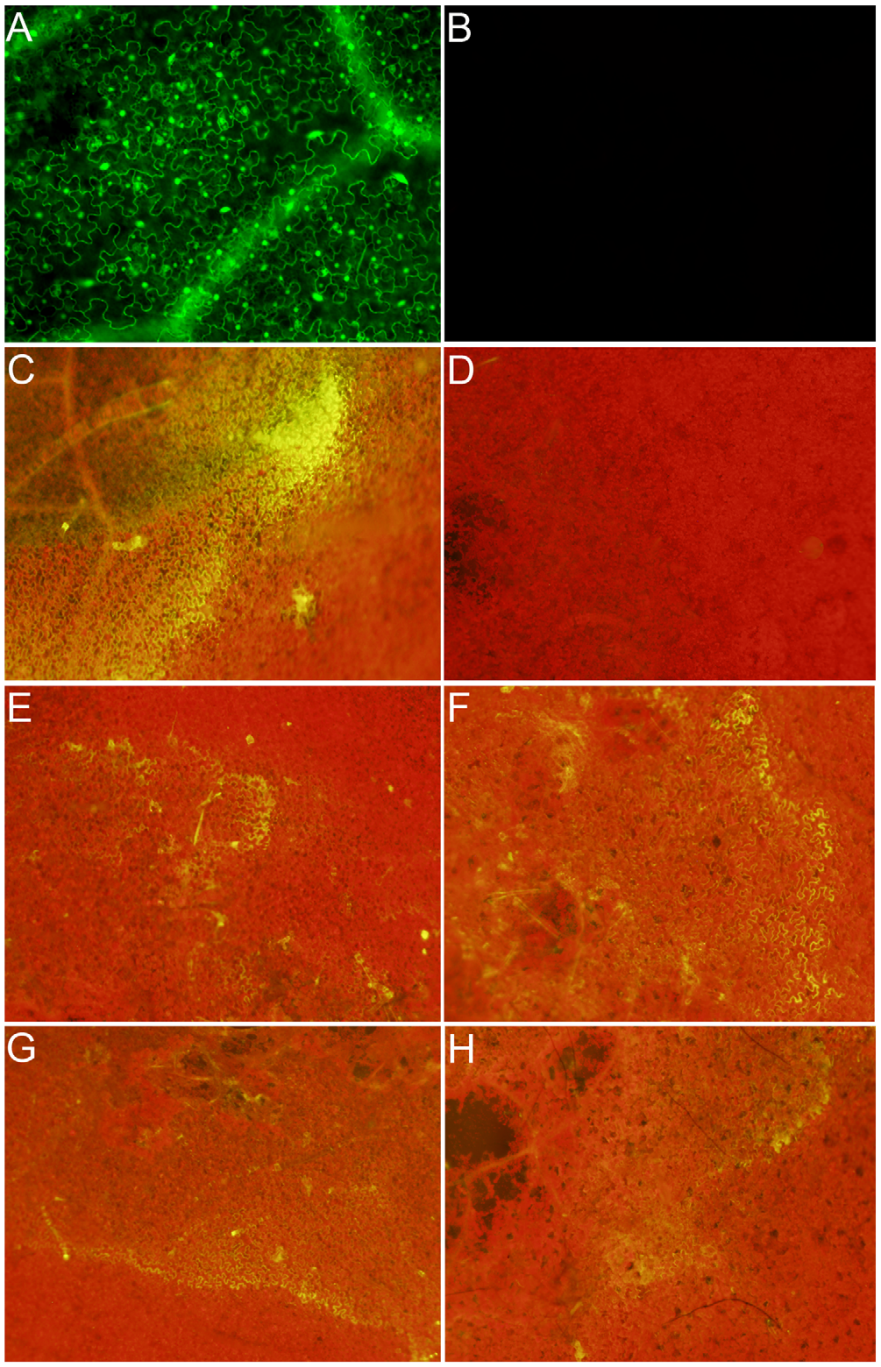
Wild-type Sclerotinia, but not the OA-deficient (A2) mutant, reduces the host cellular environment. Transgenic *Nicotiana benthamiana* leaves containing the redox sensitive GFP (roGFP) cassette were investigated by confocal microscopy using (A) 410 nm and (B) 474 nm filters for observation of roGFP under oxidizing and reducing conditions, respectively. Two lines were chosen (line 2 shown here) for further analysis and inoculated with agar plugs containing actively growing (C) Wild-type Sclerotinia, (D) oxalate deficient A2 mutant, (E) nox1 mutant, (F) nox2 mutant, (G) sod mutant, and (H) wild-type *Botrytis cinerea*. Eight hours post-inoculation leaves were visualized under a 474 nm filter for observation of the reduced form of the roGFP.

**Figure 3:**
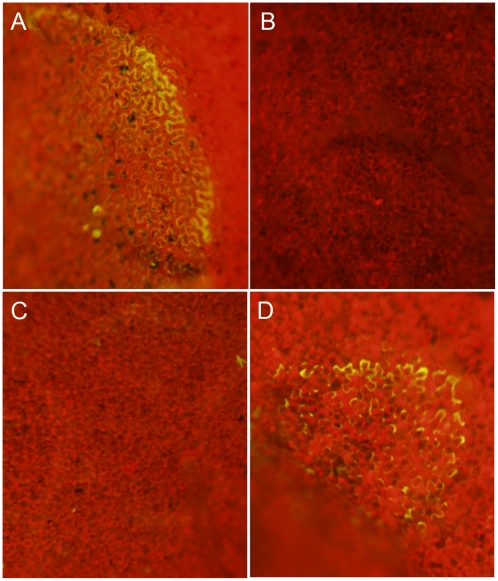
Oxalic acid modulates the host redox environment. Transgenic redox sensitive GFP *Nicotiana benthamiana* leaves from line 2 were infiltrated by a needle-less syringe with (A) 10 mM KOA pH 7, (B) 10 mM KOA pH 3, (C) 10 mM HCl, or (D) 10 mM DTT. Four hours post-infiltration, leaves were visualized under a 474 nm filter for observation of the reduced form of the roGFP.

Following these observations, we theorized that *Sclerotinia* may alter the redox environment in the host to avoid detection. To more accurately determine whether wild type *Sclerotinia* suppresses the oxidative burst by modulation of the host redox environment, we used a real-time plant-based redox sensitive GFP reporter, ro-GFP [15]. In this system, the GFP chromophore has been altered such that the excitation wavelength is influenced by the oxidation state of the environment. The GFP is constitutively expressed; however, the excitation wavelength (410 versus 470 nm) is reliant upon the redox potential of the environment. During reducing conditions the excitation occurs at 470 nm while under oxidizing conditions excitation occurs at 410 nm and thus these environments can be differentiated using appropriate filters. Transgenic *Nicotiana benthamiana* lines were generated containing a 35S driven redox sensitive GFP cassette. Initial confocal microscopy analysis supported GFP excitation at 410 nm filter but failed to detect GFP excitation and fluorescence at 474 nm (Figure 2A and 2B). This suggests that in the default state reducing conditions are not detectable. To determine whether GFP fluorescence can be observed under reducing conditions, we infiltrated leaves with the established reducing agent, DTT. DTT created a reducing environment within the cell and GFP fluorescence was successfully observed with the 474 nm excitation filter (Figure 3D).

Since GFP fluorescence was not observed in the ro-GFP plants under the 474 nm filter (excited only under reducing conditions), this filter was used to evaluate the reductive state of host cells following challenge with wild type *Sclerotinia*. The two strongest ro-GFP expressing lines (2 and 6) from the initial confocal microscopy analysis were chosen and challenged with the wild type and the OA-deficient A2 mutant strains. As expected, and consistent with our previous confocal analysis, GFP expression was not observed under the 474 nm filter prior to infection; leaves challenged with the wild type strain however revealed regions of strong fluorescence at this wavelength (Figure 2C). Notably, the fluorescent region was distinct and located in advance of fungal growth before becoming rapidly diffuse. Unlike its wild type counterpart, but consistent with the disease phenotype and ROS staining results, the A2 mutant failed to induce GFP fluorescence (Figure 2D). These results suggest that the wild type strain, but not the OA-deficient A2 mutant, is able to promote a reducing environment within the host during the initial stages of infection, possibly through the secretion of OA.

The necrotrophic fungal pathogen *Botrytis cinerea* was also examined. *B. cinerea* is closely related to *Sclerotinia* but produces lower quantities of OA; and as shown here, reduced flouerscence/reduction (Figure 2H). The role of OA in *B. cinerea* pathogenesis has been examined by several groups however, details are not clear. To further study the relationship between cellular reduction and oxalic acid production, we inoculated two NADPH oxidase (*Ssnox1*, *Ssnox2*) and a superoxide dismutase (*Sssod1*) *Sclerotinia* mutant strains generated in our lab. These mutants are altered in redox capabilities as indicated by the observation that lower levels of OA are produced compared to wild type (Figure S1 and S2). These strains generated reducing conditions in the host to levels that were intermediate between the wild type strain and the OA-deficient A2 mutant (Figure 2E–2H). The ability to reduce the host environment was related to the level of OA secreted by all of these strains. Therefore, the secretion of OA, generation of reducing conditions, and intra-/inter-cellular ROS signaling between pathogen and host appear to be integral determinants for *Sclerotinia* pathogenicity.

### *Sclerotinia*-mediated modulation of host redox status occurs via secretion of OA

To assess whether OA can modulate the host redox environment directly, we monitored the cellular redox state in OA infiltrated leaves. The acidification of cells is known to dampen the oxidative burst [14], therefore to examine acidification as a factor we used the OA salt, KOA, buffered to pH 3 and pH 7. We included 10 mM HCl as an additional control. *Nicotiana benthamiana* ro-GFP leaves from lines 2 and 6 were infiltrated with 10 mM KOA (pH 3 and 7) and 10 mM HCl, (10–20 mM OA is commonly found in diseased plant tissue [7]). Consistent with previous reports showing OA induced host PCD was independent of acidification [12], infiltration of KOA at pH 7, but not pH 3, was able to reduce the host cellular environment and support GFP fluorescence under the 474 nm excitation filter (Figure 3A and 3B). Further confirmation that OA induced reducing conditions are independent of its ability to acidify the host cellular environment was supported by HCl treatment, which also failed to induce GFP fluorescence (Figure 3C). These results strongly correlate with our previous studies [12] that show induction of ROS and PCD occur independent of the acidification ability of OA and demonstrate that OA alone is sufficient for mediating reducing conditions in plant cells. Taken together, we suggest that OA suppresses the oxidative burst, at least in part via the generation of reducing conditions and subsequently triggers ROS induced DNA fragmentation and PCD at neutral (pH 6–7) but not acidic (pH 3) conditions. Our results provide further evidence supporting the importance of OA as a key *Sclerotinia* pathogenicity factor.

### Chemical reduction of the host environment is sufficient to aid *Sclerotinia* infection

**Figure 4:**
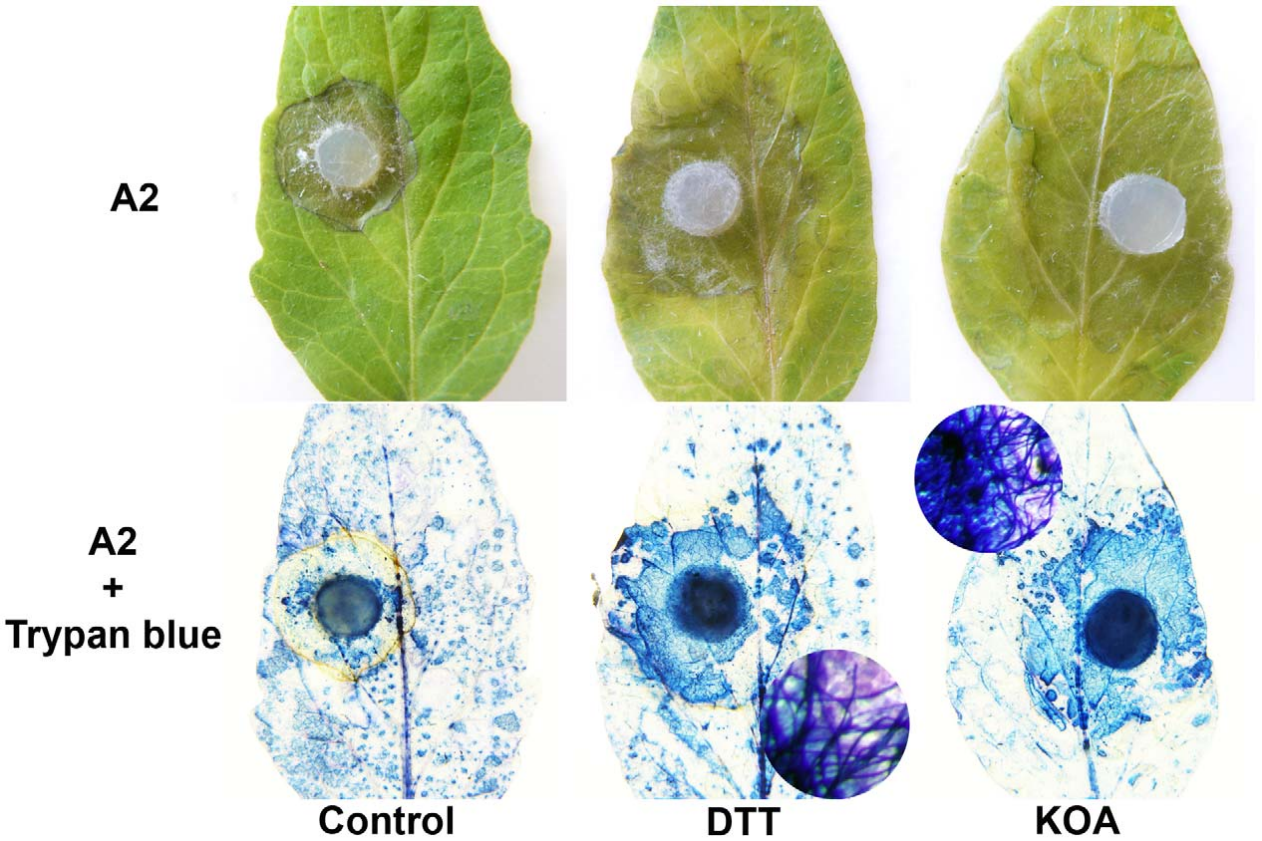
Artificial induction of reducing conditions reverts the A2 phenotype. Tomato leaves were pre-infiltrated with H_2_0 (left), and either 10 mM KOA pH 7 (right) or 10 mM DTT (middle). The H_2_O, KOA, and DTT infiltrated regions were inoculated with agar plugs containing actively growing OA-deficient A2 strain (top panel). Additionally, trypan blue was used to stain fungal mycelia within the infiltrated area (bottom panel). The circles represent a close up of the stained area showing trypan blue stained mycelia.

If the induction of a reduced state in the cell via secretion of OA is a necessary and sufficient component of *Sclerotinia* pathogenicity, we reasoned that pathogenicity of the OA-deficient (A2) mutant could be enhanced via the induction of an “artificial” reducing environment. Exogenous application of DTT (and KOA) enhanced disease development of the OA-deficient A2 mutant (Figure 4). Trypan blue staining of fungal tissue verified that the A2 mutant can now grow within the DTT and KOA infiltrated areas (Figure 4). Thus, these observations are consistent with the premise that *Sclerotinia* induces disease by initially triggering reducing conditions in the cell during the early stages of infection. Furthermore, these data also show that the ability of the A2 mutant to cause disease is restored under these conditions. The non-pathogenic phenotype associated with OA deficiency is due, at least in part, by the inability to create a reducing environment in the host. Taken together, these data suggest that the host redox environment, specifically cellular reduction, is an integral pathogenicity component of *Sclerotinia* and may contribute to the broad host range displayed by this necrotrophic fungus.

### *Sclerotinia* rapidly creates reducing conditions prior to oxidative stress and plant cell death

**Figure 5:**
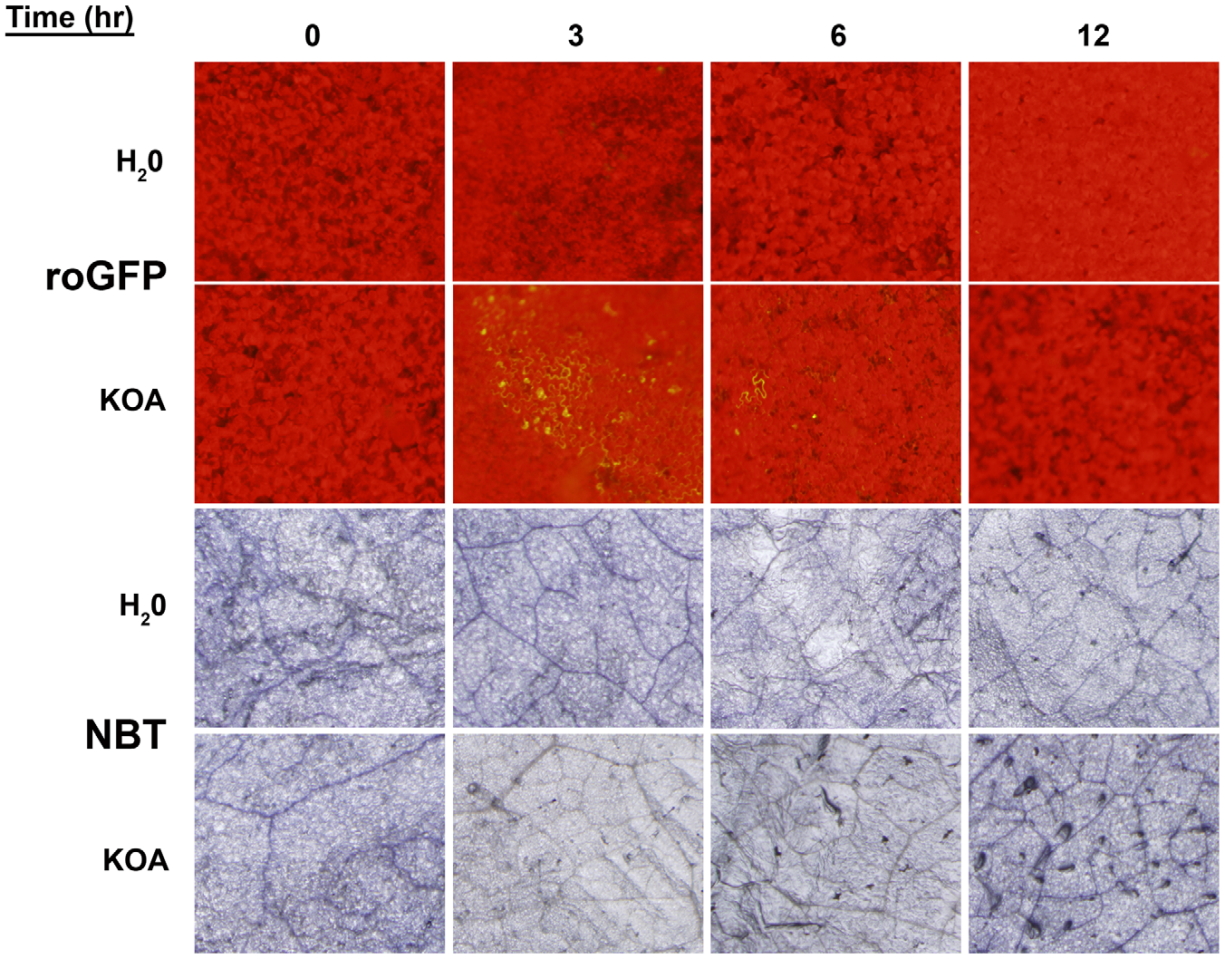
Oxalic acid induces reducing conditions that correlates with the dampening of the host oxidative burst. Detached leaves of roGFP plants were infiltrated with either H_2_O or 10 mM KOA buffered to pH 7, and were examined for GFP fluorescence (Top two panels) over a time-course (0, 3, 6, and 12 hours). The same leaves were also stained for superoxide production (Bottom two panels) with nitro-blue tetrazolium (NBT).

A conundrum of the *Sclerotinia*/OA system as noted by our previous experiments, is that OA suppresses the generation of host plant ROS [5], but is also capable of inducing plant ROS during disease development culminating in PCD [12], both of which are necessary for pathogenesis. Based on the available evidence, we hypothesize that OA triggers a rapid, but transient reduced state in the cell that is temporally followed by oxidation leading to host cell death and disease. To investigate this possibility, we infiltrated leaves from intact plants with water or KOA buffered to pH 7, and examined GFP fluorescing regions over a 12 hour time course, sampled every three hours. Additionally, we stained the same leaves for superoxide production with nitro-blue tetrazolium (NBT). Consistent with our earlier results, infiltration of KOA at pH 7 induced reducing conditions and supported GFP expression in leaf cells three hours post-infiltration (Figure 5). OA-mediated reduction and excitation of ro-GFP (474 nm) was also observed six hours post-infiltration but at lower levels than those observed three hours-post infiltration (Figure 5). GFP excitation was not observed in any of the water infiltrated controls or with KOA 12 hours post-infiltration. The observation of reducing conditions as early as three hours post-infiltration of OA suggests that *Sclerotinia* rapidly induces strong reducing conditions during the initial stages of infection. This correlates with the dampening of the host oxidative burst, and provides further evidence that *Sclerotinia* “prepares” host cells for infection via the induction of a reducing environment.

In contrast to GFP expression, NBT staining detected low levels of superoxide at 0 hours post-infiltration, however, this also occurred for the water control and is more likely a result of injury during the infiltration process rather than elicitation of oxidizing conditions. In comparison to the water controls, ROS levels in the KOA infiltrated samples were reduced 3 hours post-infiltration. These results are in agreement with the ro-GFP time-course that demonstrated reducing conditions in leaf cells as early as three hours post-infiltration (Figure 5). There were slight differences in the ROS levels between the water controls and KOA samples 6 hours post-inoculation. However, 12 hours post-infiltration, there was a consistent and reproducible increase in NBT staining for the KOA infiltrated sample in comparison to the water control. These observations are in accordance with our previous studies that showed strong DAB staining in leaves 24 hours post OA treatment [12]. The NBT-staining results suggest that following the initial OA induced reducing environment, oxidizing conditions prevail in the cell. We have previously shown that direct OA treatment of plant tissue induces ROS and plant cell death; both of which can be inhibited chemically [12].

Thus, OA secreted by *Sclerotinia* appears to have dual opposing functions. During the early stages of infection, reducing conditions are induced that may suppress the oxidative burst, host defenses, and possibly other host processes. Once infection is established however, oxidizing conditions are generated in response to the fungus and cell death occurs. In this manner *Sclerotinia* uses OA as a signaling molecule to control the direction of the host redox environment, plant defense responses, and cell death pathways.

## Discussion

Traditionally, necrotrophs were thought to directly kill host tissue via the secretion of toxins and degradative enzymes. Recent studies with *Cochliobolus*, *Botrytis* and *Sclerotinia* however, suggest that the infection process may be more subtle than originally believed and that certain necrotrophs do not kill the cell directly but instead commandeer plant PCD pathways for their own benefit. Although the precise mechanism by which these pathogens control host PCD is unknown, emerging evidence from *Sclerotinia* suggests that ROS plays a significant role. As an intermediary of PCD responses, low concentrations of ROS function as signaling molecules during pathogen development [16] and during pathogen-host interactions [17]. In this study we used a plant-based redox sensing GFP expression system, histological staining and reverse fungal genetics to demonstrate a role for oxalate in the mediation of the host redox environment and preparation of host cells for *Sclerotinia* infection.

Cells have a limited number of molecules and combinations that can be deployed for various aspects of regulation, growth and development. The use of a single molecule to perform several seemingly unrelated tasks is a common strategy employed by organisms to increase the range of functions using a defined and limited repertoire of molecules. For example, the catabolic enzyme mannitol dehydrogenase is also a pathogenesis related (PR) protein that can be induced by pathogens even in mannitol non-containing plants [18]. Proline is a non-essential amino acid that functions as an osmolyte during stress. We have shown that proline is also a potent anti-oxidant and is associated with such far ranging stresses from mammalian diseases to plant drought responses as well as nutrition [19]. In this study we show that fungal oxalic acid is another such example. This “simple” organic (dicarboxylic) acid, is efficiently used by *Sclerotinia* for a range of processes that include, direct toxicity, development (sclerotia), pH signaling, activation of cell wall degrading enzymes, plant guard cell regulation, chelation of calcium, vascular plugging, elicitation of programmed cell death [8], [9], [reviewed in 10], [12] and in this report, directing the redox environment in the host cell for pathogenesis. Our accumulating evidence shows that OA generates reducing conditions in the cell, correlating with the inhibition of the host oxidative burst and other defense responses. This is followed by an OA mediated plant programmed cell death and eventual establishment of disease.

Several lines of evidence are consistent with these conclusions: 1) When OA is not present in the “infection court” as is the case with the A2 strain, the plant oxidative burst is clearly evident and a resistant response ensues 2) *Sclerotinia* rapidly induces reducing conditions in host cells in advance of fungal growth, and this redox manipulation is tightly associated with the onset of disease. 3) The modulation of the host redox environment is subverted by OA; OA-deficient mutants were unable to induce reducing conditions and were unable to cause disease. 4) Temporally, this reduction precedes ROS synthesis which is necessary for cell death. 5) The optimum pH for OA induced reduction corresponds to the pH conditions required for OA induced ROS and subsequent PCD. 6) The addition of a potent reducing agent, DTT, generates transient reducing conditions during initial infection that suppress host defense and the oxidative burst to revert the OA-deficient mutant phenotype and restore pathogenicity. Therefore OA appears to have dual opposing roles in *Sclerotinia* pathogenesis; OA initially inhibits ROS-mediated plant defense responses, but later promotes ROS generation in the plant followed by programmed cell death. These data address a long-standing issue in this system involving the requirement for *Sclerotinia*/OA to both inhibit and promote ROS to achieve pathogenic success.

In *Monilinia fructicola*, a stone fruit pathogen related to *Sclerotinia*, intracellular antioxidant levels in the fungus are influenced by host derived phenols, altering the fungal redox environment, though not affecting fungal growth. However pathogen gene expression and pathogen infection structure differentiation were directly affected and were related to changes in electrochemical redox potential. *Monilinia* also possesses a redox regulated cutinase gene, which is upregulated during oxidative stress and when overexpressed, increases virulence [20], [21]. Thus, the redox balance in both the host and pathogen can be a key battlefield in determining the outcome following pathogen challenge.

The mechanism by which OA triggers such conditions is a key question, and could involve redox molecules such as thioredoxins. Thioredoxins are ubiquitous redox proteins that act as antioxidants by facilitating the reduction of proteins involved in a variety of physiological roles within cells including the activation of plant defense pathways. For example, the redox-sensitive *Arabidopsis* thioredoxin-5 (TRX5) mediates a conformational change in the non-expressor of PR genes (NPR1), which is necessary for the activation of plant immunity [22]. NPR1 is a key transcriptional regulator in the signaling pathways that lead to systemic acquired resistance [23]. In unchallenged plants, NPR1 is maintained as an inactive oligomer in the cytoplasm via redox-sensitive disulphide bonds. During pathogen challenge however, the redox state of the cell is altered via the plant hormone, salicylic acid. This change in cellular redox leads to the reduction of disulphide bonds and release of an NPR1 monomer that translocates to the nucleus where it functions as transcription factor for defense signaling. Although the outcome is different to that observed during *Sclerotinia* interactions, the overall strategy of host redox alteration during cell death regulation (in this case plant immunity) is maintained. In the case of *Sclerotinia*, the pathogen is in control of the redox environment and cell death pathways; the oxidative burst does not occur and the pathogen directly benefits. In contrast, during an immune response, the plant controls the redox environment and cell death pathways to the detriment of the pathogen. As observed following inoculation with the A2 mutant, the host was able to mount an oxidative burst, as well as callose deposition and thus effectively resist infection by mounting an HR-like response.

Another potential link between thioredoxins and pathogenicity involves the necrotrophic oat pathogen, *Cochliobolus victoriae* that produces the host selective toxin victorin. Analogous to *Sclerotinia*, victorin deficient *Cochliobolus* strains are non-pathogenic on susceptible oat genotypes; exogenous application of victorin results in disease symptoms, including features associated with apoptotic-like programmed cell death such as DNA laddering and caspase-like protease activity [24]. Of relevance to our work, this study has shown that sensitivity to victorin also requires the activity of the plant thioredoxin, TRX5. In this case, victorin may recruit TRX5 to alter the host cellular environment. Although the exact role for TRX5 activity during sensitivity to victorin is unknown, the observation that victorin sensitivity requires thioredoxin activity suggests that pathogen mediation of the host redox environment may also occur during *C. victoriae* challenge. Further evidence supporting the key role of host redox manipulation for optimal *Sclerotinia* infection is illustrated by work with oxalate oxidases. Oxalate oxidases (“germin” proteins) are members of the oxidoreductase family found in all monocots and catalyze the breakdown of OA into CO_2_ and H_2_0_2_ [25]. Knowing that OA is a pathogenicity determinant of *Sclerotinia*, several groups have generated dicot plants over-expressing a monocot oxalate oxidase and observed increased resistance to *Sclerotinia* [25]–[27]. Similarly, our studies also demonstrated high levels of plant H_2_0_2_ and delimited growth in response to infection with the oxalate deficient A2 mutant. Therefore, removal of OA during infection, either by expression of an oxalate oxidase in the plant or genetically, through the use of OA-deficient *Sclerotinia* mutant strains (i.e. A2) leads to oxidizing conditions controlled by the host and the mounting of host defense responses. Additionally, *Sclerotinia*, while possessing an impressively broad host-range, does not infect monocots. We speculate that this inability to create reducing conditions in the host explains at least in part, why *Sclerotinia* diseases are essentially limited to dicotyledonous plants. In future studies we will identify monocot germin knockout lines and evaluate pathogenic behavior of *Sclerotinia*.

The host target(s) of OA are not known. Recently, we have found that *Arabidopsis* plants lacking BIK1, were significantly enhanced in susceptibility to A2 (Figure S3). BIK1 is an *Arabidopsis* cytoplasmic receptor kinase (Botrytis induced kinase) that mediates crosstalk between defense pathways in *Arabidopsis* [28]. BIK1 was originally discovered as a component in plant defense against the necrotroph *Botrytis cinerea* [29] and has subsequently been shown to play an important role in defense signaling to initiate MAMP triggered immunity [28], [29]. Intriguingly, our initial studies have also shown that OA can modulate the phosphorylation status of BIK (data not shown). Studies are in progress to determine whether this is a direct or indirect interaction, but regardless, OA appears to inhibit defense signaling mediated by BIK. Thus, BIK represents a potential host target for *Sclerotinia*. Moreover there may be shared components of MAMP defense signaling and necrotrophic fungal pathogenesis.

In summary, we show in real-time using a redox sensitive GFP reporter that the earliest detectable host response to *Sclerotinia* challenge is the creation of a fungal (OA) induced reducing environment that is observed in advance of pathogenic fungal growth. In contrast, the loss of oxalate in the fungus leads to the failure of host colonization and induces strong plant defense responses, as noted with the OA-deficient A2 mutant. This strain failed to colonize the host and induced strong HR-like host defenses (including an oxidative burst) similar to those commonly observed during incompatible biotrophic infections. We suggest that reducing the cellular environment directly or indirectly suppresses the host-plant oxidative burst and defense mechanisms, including callose formation. The net result provides *Sclerotinia* with precious time for unimpeded establishment in host tissue and the hijacking of host pathways to generate ROS and induce PCD; a perfect environment for this necrotroph. Thus the initial pathogenic phase of this well established necrotroph surprisingly, displays features similar to those observed during compatible biotrophic or early stage hemi-biotrophic interactions.

## Materials and Methods

### Plant and fungal materials

*S. sclerotiorum* isolate 1980 and an oxalate-deficient mutant (A-2) of this strain were maintained at 24°C on potato dextrose agar as previously described by [7]. NOX1, NOX2, and SOD strains were generated for a different study using a split-marker-deletion approach as described by [30]. *Botrytis cinerea* strain B05.10 was provided by Dr. Jan van Kan. The c-roGFP1 was kindly donated by Lewis Feldman. Wild type and transgenic *Nicotiana benthamiana* plants expressing the ro-GFP constructs [15] were generated as described below and maintained in tissue culture under a 16-h light period.

### Stable transformation of *Nicotiana benthamiana* plants using *Agrobacterium tumefaciens*

Electro-competent *Agrobacterium* (strain LBA 4404) were transformed with plasmids c-roGFP1 [15] by electroporation using an EC100 electroporator (Thermo EC) based on the method of [31]. Wild type *Nicotiana benthamiana* leaf discs were transformed by *Agrobacterium* as described by [32]. Following transformation, leaf discs were sub-cultured every two weeks on MS104 media containing timentin (200 mg/L) and hygromycin (50 mg/L). After five weeks of culture, shoots of a suitable size were transferred to MSO media containing timentin (200 mg/L) and hygromycin (50 mg/L). Replicates were generated by nodal cutting and culture on MSO media containing timentin (200 mg/L) and hygromycin (50 mg/L).

### Confocal microscopy

Emerging leaves of wild type and ro-GFP expressing *Nicotiana benthamiana* were exicised and prepared on microscope slides in distilled water. Redox GFP excitation and fluorescence were viewed using an Olympus IX81 microscope with long and short pass GFP filter sets, under a 10× magnification.

### Fungal challenge

Newly emerging leaves of wild type and ro-GFP expressing *benthamiana* were excised and inoculated with 5 mm PDA plugs containing actively growing wild type *Sclerotinia* isolate 1980, OA-deficient (A2) mutant, NaDPH oxidase 1 and 2 (nox 1 and 2) and superoxide dismutase (SOD) mutant *Sclerotinia*. Leaves were analyzed over an eighteen hour time-course for GFP fluorescence using an Olympus SZ×10 and mGFPA long pass filter set (exc. 460–490 nm, emm, 510 nm). *Botrytis cinerea* strain B05.10 was grown on Malt Agar, inoculation and GFP fluorescence analysis was performed as described above.

### Oxalic acid treatment

Newly emerging cyt-roGFP leaves were infiltrated with either 10 mM KOA buffered to pH 3 or 7, or 10 mM HCl and analyzed over an eight hour time course for GFP fluorescence using an Olympus SX-10 and the mGFPA filter set.

### Histological assays

#### Callose

Aniline blue staining was performed as described by Asselbergh and Höfte [33]. Briefly, two days post-inoculation tomato leaves were incubated in lactophenol for 60 min at 65°C, replacing lactophenol with fresh solution after 30 min. Samples were then transferred to room temperature and incubated for a further 12 hrs, washed in 50% ethanol for 5 min, and stained for 30 min in the dark with 0.01% aniline blue in 150 mM K_2_HPO_4_ (pH 9.5). Stained samples were observed under Olympus IX-81 microscope.

#### DAB staining

For detection of H_2_O_2_, eight hours post-inoculation leaves were stained in 2 mg/ml 3′3-diaminobenzidine-tetrahydrochloride (DAB) for 2–4 hr and then distained with 70% ethanol at 70°C.

#### Trypan Blue Staining

Post-infection, *Sclerotinia* challenged leaves were excised, stained with 0.05% Trypan blue for 45 min at 25°C and washed with PBS. All samples were observed for blue staining using white light and an Olympus SZ×10 microscope.

### Oxalic time-course assay

For investigation of OA-mediated reduction, oxidation and PCD, newly emerged roGFP leaves were infiltrated with either 10 mM KOA buffered to pH 7 or water as described above. Infiltrated leaves were excised at time points of 0, 3, 6 and 12 hours post-treatment and analyzed for GFP expression using a Olympus SX-10 microscope and a mGFPA long pass filter set (exc. 460–490 nm, emm, 510 nm), DAB staining and Evans blue staining, respectively.

## Supporting Information

Figure S1 **Oxalate level measurements in wild-type, sod and A2 mutant strains.** Oxalic acid concentrations in the wild-type 1980 strain and the derived sod and A2 mutant strains were determined using an oxalate detection kit according to manufacturer's recommendations.
(TIF)

Figure S2 **Oxalate level measurements in wild-type, nox1, nox2 and A2 mutant strains.** Oxalic acid concentrations in the wild-type 1980 strain and the derived nox1, nox2, and A2 mutant strains were determined using an oxalate detection kit according to manufacturer's recommendations.
(TIF)

Figure S3 **Arabidopsis** ***bik1*** **mutant plants are susceptible to the OA-deficient mutant A2.** *Arabidopsis* wild type and *bik1* mutant leaves were inoculated with agar plugs containing actively growing OA-deficient A2 strain.
(TIF)
