# Supplementary figures and images for: Tipping the Balance: Sclerotinia sclerotiorum Secreted Oxalic Acid Suppresses Host Defenses by Manipulating the Host Redox Environment
Source: PLoS Pathog. 2011 Jun 30;7(6):e1002107. doi: 10.1371/journal.ppat.1002107 (PMC3128121; doi:10.1371/journal.ppat.1002107)

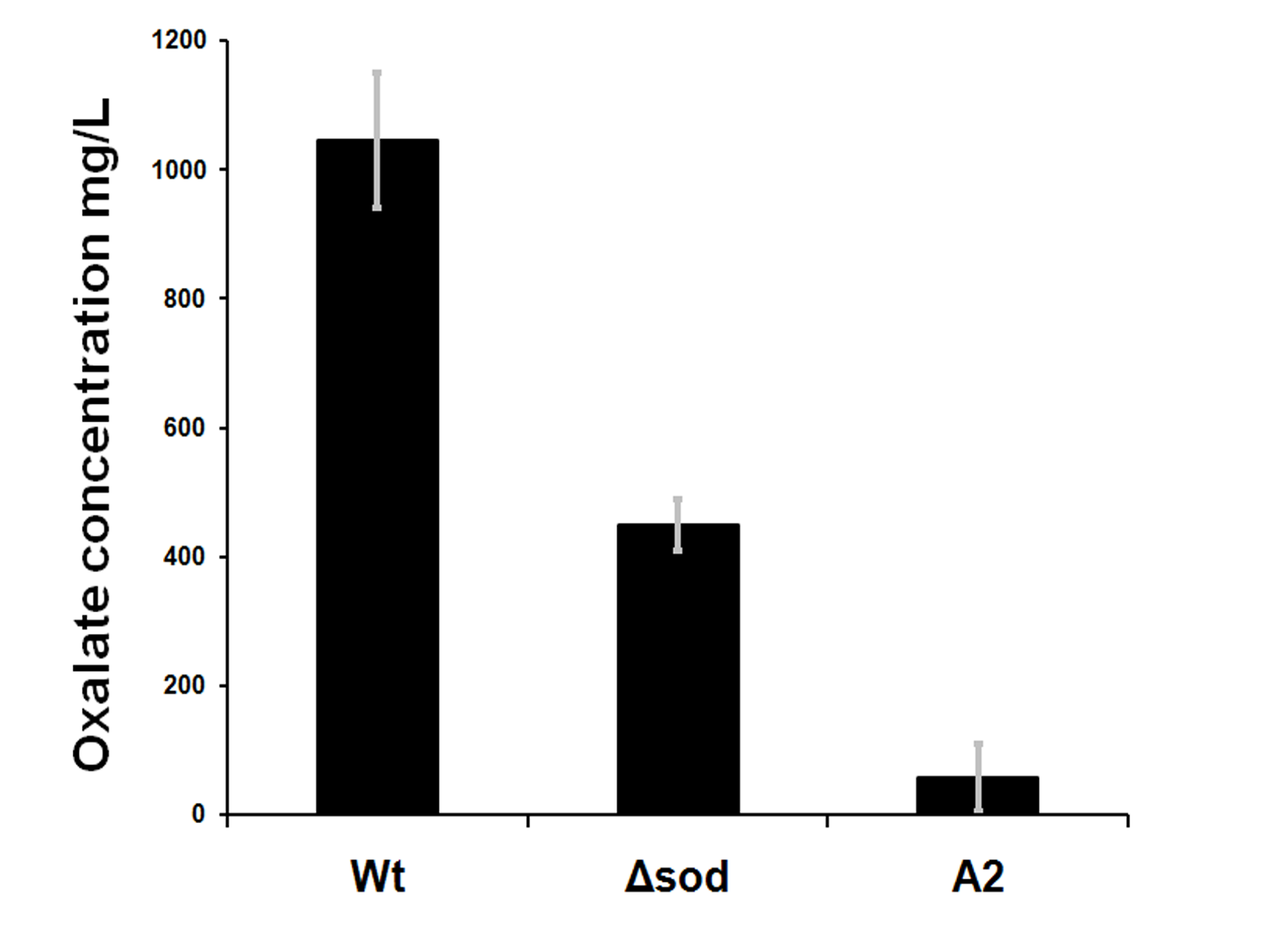

Supplement: Figure S1 — Oxalate level measurements in wild-type, sod and A2 mutant strains. Oxalic acid concentrations in the wild-type 1980 strain and the derived sod and A2 mutant strains were determined using an oxalate detection kit according to manufacturer's recommendations. (TIF) [file ppat.1002107.s001.tif]

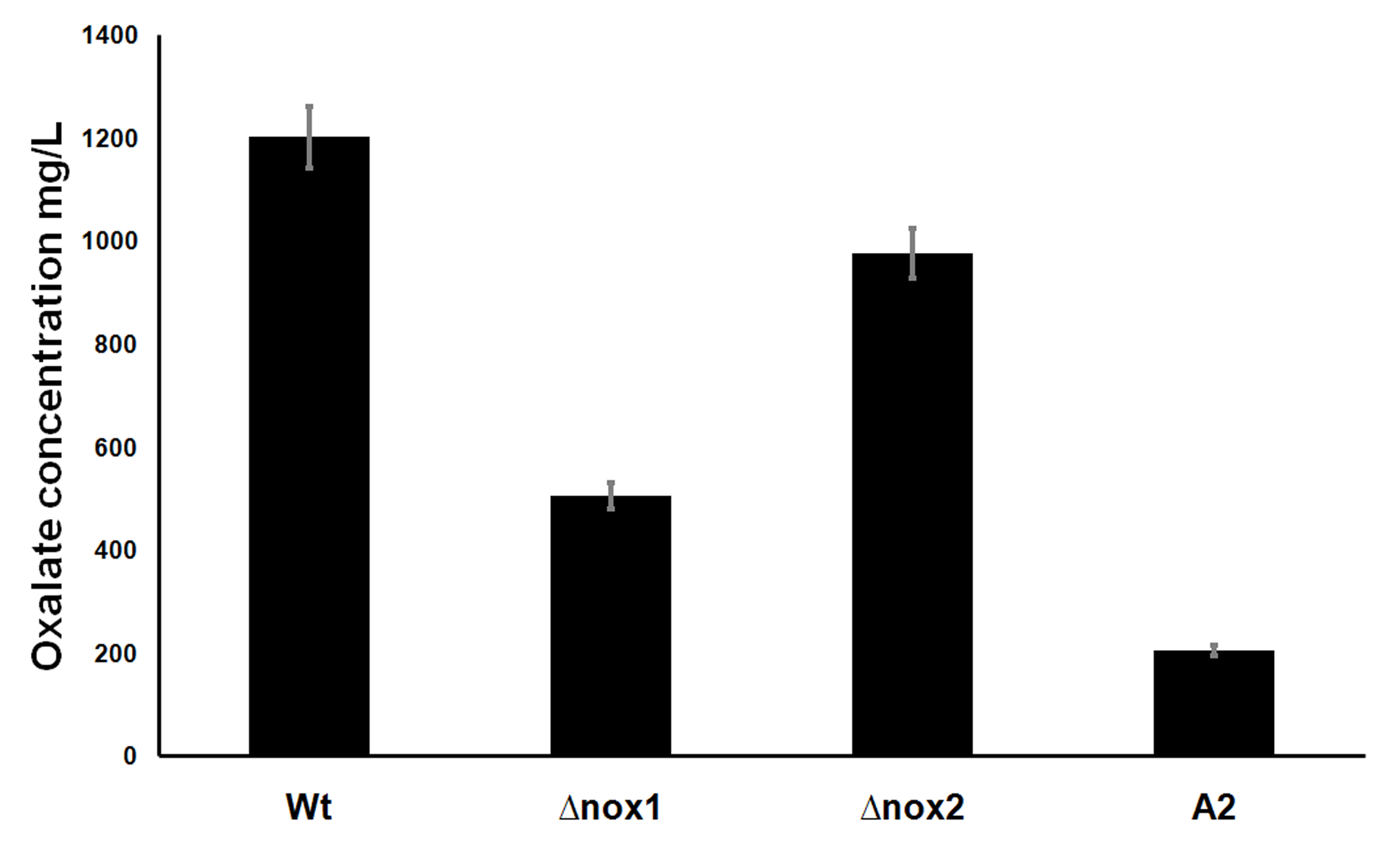

Supplement: Figure S2 — Oxalate level measurements in wild-type, nox1, nox2 and A2 mutant strains. Oxalic acid concentrations in the wild-type 1980 strain and the derived nox1, nox2, and A2 mutant strains were determined using an oxalate detection kit according to manufacturer's recommendations. (TIF) [file ppat.1002107.s002.tif]

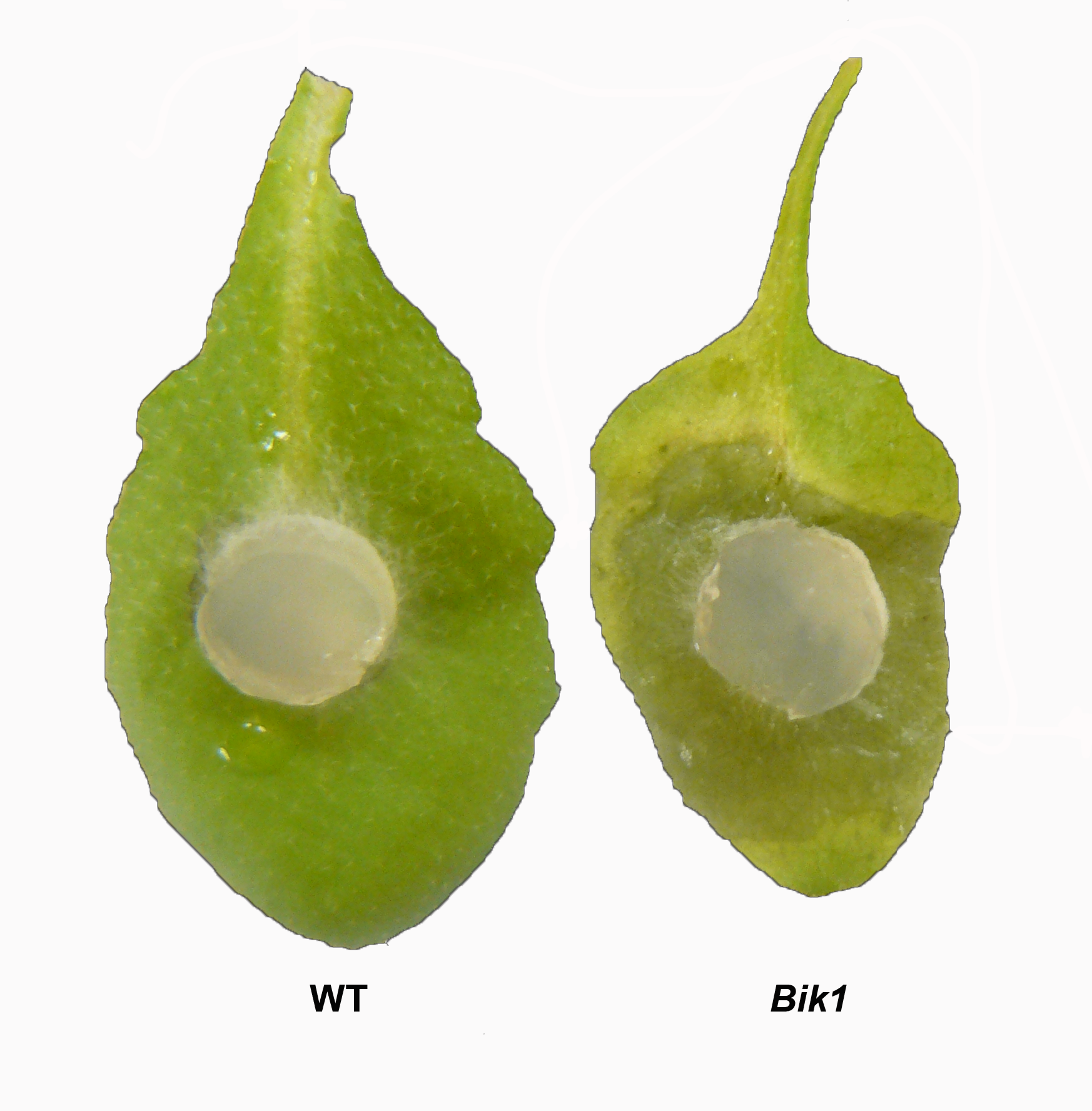

Supplement: Figure S3 — Arabidopsis bik1 mutant plants are susceptible to the OA-deficient mutant A2. Arabidopsis wild type and bik1 mutant leaves were inoculated with agar plugs containing actively growing OA-deficient A2 strain. (TIF) [file ppat.1002107.s003.tif]
